# Development of a Shoulder Disarticulation Prosthesis System Intuitively Controlled With the Trunk Surface Electromyogram

**DOI:** 10.3389/fnbot.2020.542033

**Published:** 2020-10-29

**Authors:** Susumu Kimizuka, Yohei Tanaka, Shunta Togo, Yinlai Jiang, Hiroshi Yokoi

**Affiliations:** ^1^The University of Electro-Communications of Graduate School of Informatics and Engineering, Chofu, Japan; ^2^JR Tokyo General Hospital, Tokyo, Japan; ^3^Beijing Institute of Technology of Beijing Advanced Innovation Center for Intelligent Robot and Systems, Beijing, China

**Keywords:** multipoint measurement, principal component analysis, shoulder disarticulation prosthesis, electromyogram, shoulder amputee

## Abstract

We developed an intuitively operational shoulder disarticulation prosthesis system that can be used without long-term training. The developed system consisted of four degrees of freedom joints, as well as a user adapting control system based on a machine learning technique and surface electromyogram (EMG) of the trunk. We measured the surface EMG of the trunk of healthy subjects at multiple points and analyzed through principal component analysis to identify the proper EMG measurement portion of the trunk, which was determined to be distributed in the chest and back. Additionally, evaluation experiments demonstrated the capability of four healthy subjects to grasp and move objects in the horizontal as well as the vertical directions, using our developed system controlled via the EMG of the chest and back. Moreover, we also quantitatively confirmed the ability of a bilateral shoulder disarticulation amputee to complete the evaluation experiment similar to healthy subjects.

## Introduction

Upper-limb prostheses are wearable instruments that are used to reconstruct the lost exercise function and appearance of upper limbs (Bandara et al., 2012). Among these, the prosthesis corresponding to the shoulder defect is known as the shoulder disarticulation prosthesis (ISO9999 06.18.18). A majority of these shoulder disarticulation prosthesis enable the user to manually control the elbow and hand movements via the wire, which is known as body-powered shoulder disarticulation prosthesis. However, the range of motion and muscular strength of the user limits its performance. Additionally, a sufficient grasping force is necessary to stably hold the items used in daily life. Therefore, the body-powered shoulder disarticulation prosthesis has high user expectation.

By contrast, the powered shoulder disarticulation prosthesis operates using an actuator such as a motor, and its control is generally achieved via electromyogram (EMG) (Bandara et al., 2012). The powered shoulder disarticulation prosthesis introduces less physical burden on the user as compared to the body-powered shoulder disarticulation prosthesis, to assist the external force. Moreover, by mounting multiple actuators, it also allows the user to operate multiple degrees of freedom from the shoulder to the hand (José-Alfredo et al., 2018; Osmar et al., 2019). However, increasing the actuators and motion patterns requires many several control signals, and obtaining these signals method is difficult, because the position of obtaining the muscle potential is limited by the shoulder detachment condition of the user.

Previous studies proposed the estimation of motion intention of the user through the sole pressure, tongue movement, and voice (Carrozza et al., 2005; Johansena et al., 2012; Syeda et al., 2018). Additionally, the targeted muscle reinnervation (TMR) method was proposed to transfer nerves connected to the lost arm to the muscle of the trunk surgery, and to estimate the user intention to operate using the muscle potential (Johns Hopkins Unveils Proto 2, 2007; DARPA's “Luke Arm” Readies for clinical trials, 2011; Les, 2014). The former method can easily obtain control signals; however, it is unable to completely control the prosthetic hand intuitively. Contrarily, the latter method is suitable for controlling an intuitive prosthetic hand that is indirectly controlled by the arm nerve through the myograph. However, this latter approach requires a few months for surgery and rehabilitation (Kuiken et al., 2004; Cheesborough et al., 2015), and the burden on users could be significant. Other related studies used electrocorticogram signals to control the robot arm like a shoulder disarticulation prosthesis (Yanagisawa et al., 2011; Leigh et al., 2012). However, it was also necessary to implant electrodes in the skull through surgery, which puts a heavy burden on users. Therefore, it is necessary to develop a powered shoulder disarticulation prosthesis system that can be used intuitively without requiring long-term training and surgery.

The purpose of this study is to develop an intuitively operational powered shoulder disarticulation prosthesis (myoelectric shoulder disarticulation prosthesis) that can be implemented without the use of surgery and can also be used without long-term training, while being controlled by a trunk electromyogram. In this study, we used the word “intuitive” to describe the state in which the users can understand the relationship between their motions and the myoelectric shoulder disarticulation prosthesis without long-term training. In addition, “intuitive operation” is defined as flexion and extension movements of the shoulder disarticulation prosthesis controlled by using relevant myoelectric information of the flexion and extension movements of users. The proposed shoulder disarticulation prosthesis aims to grasp and transfer objects, which is considered as the minimum body functional requirement in daily life. Methods used to realize these functions with simple a mechanism are described in Chapters 2 to 3. Chapter 2 describes the lightweight myoelectric shoulder disarticulation prosthesis, which is operated by the shoulder and elbow only in the sagittal plane developed for achieving the goal. Additionally, it also describes the control method based on pattern recognition technique (Kato et al., 2006) to control the shoulder disarticulation prosthesis without long-term training. Moreover, the specific method of identifying the measurement sites of EMG suitable for the control of myoelectric shoulder disarticulation prosthesis is described. Chapter 3 describes the task of grasping and moving objects to evaluate the performance of our developed system.

## 4-DoF Shoulder Disarticulation Prosthesis System

**Figure 1:**
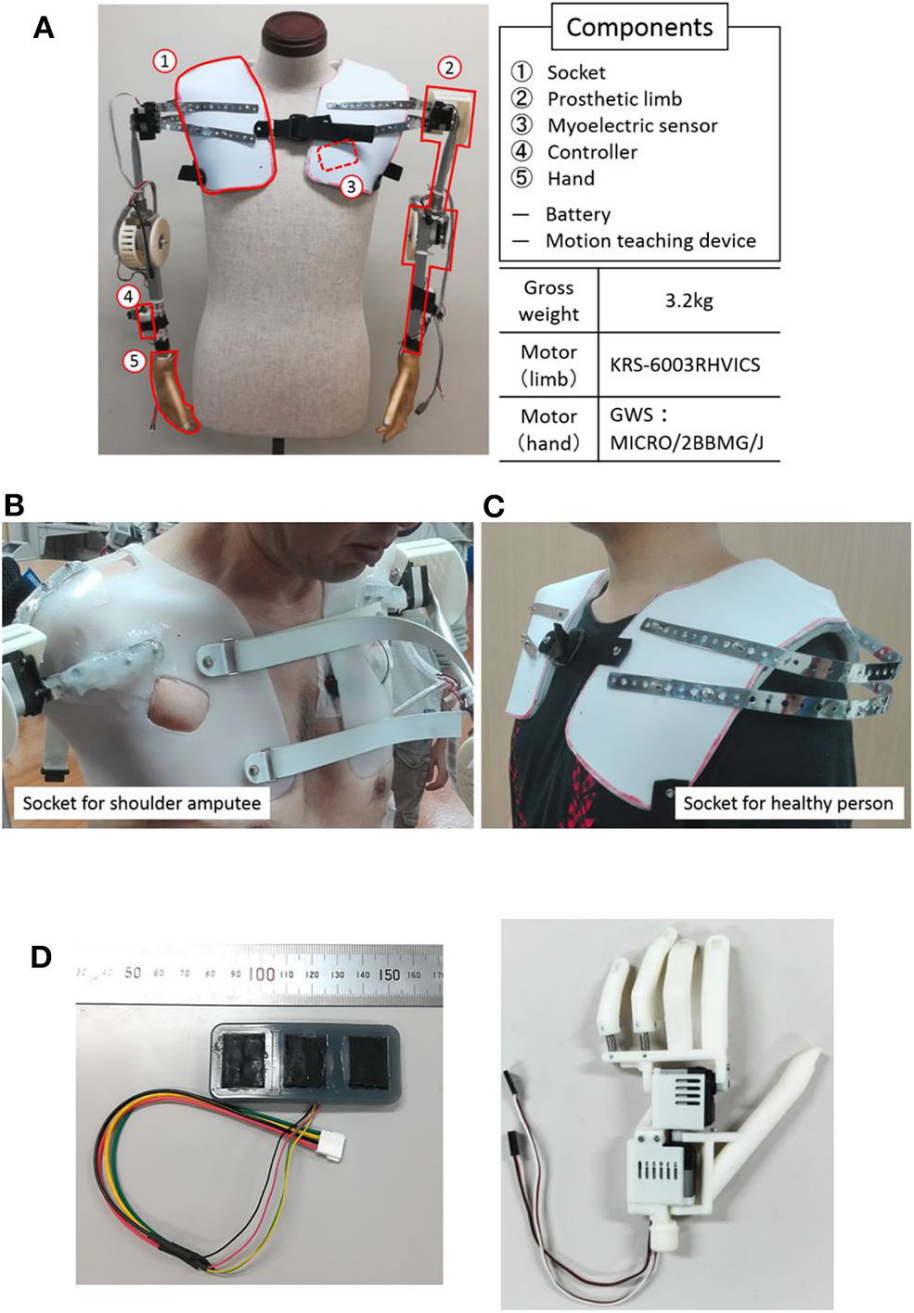
System overview and components. **(A)** An overall view of the proposed EMG shoulder disarticulation prosthesis system. **(B)** Socket. **(C)** Myoelectric sensor. **(D)** Hand.

Figure 1 shows the overall view of our developed myoelectric shoulder disarticulation prosthesis system. As mentioned in the previous chapter, the proposed myoelectric shoulder disarticulation prosthesis is a simplified myoelectric shoulder disarticulation prosthesis equipped with the function to grasp and move objects, and to perform pattern recognition-based intuitive control, using the trunk EMG. The myoelectric shoulder disarticulation prosthesis has a servo motor built in the shoulder, elbow, and hand (thumb and four fingers), while its degree of freedom is 4. The constituent elements and the mechanical control method of the proposed system are described as follows.

### Component of System

1. Socket: The socket fixes the myoelectric shoulder disarticulation prosthesis to the upper body. In this study, we used a socket for bilateral shoulder disarticulation amputee who took the stump end mold of both shoulders made by the professional prosthetic orthotist (Figure 1B Left), and a socket for a healthy person (Figure 1B right). The weight of the socket is about 350 g on each side.
2. Arm: The arm refers to the part from the socket to the wrist, which is equipped with a direct motor driving mechanism placed on the shoulder and elbow (KRS-6003 RHVICS, KONDO, Japan). The lengths of the upper arm (linear distance between motor shafts of the shoulder and the elbow) and the forearm (linear distance between the motor shaft of the elbow to the wrist) were both 23 cm, and an aluminum pipe was used as the material. The weight is ~750 g on each side.
3. Myoelectric sensor: The myoelectric sensor shown in Figure 1C consists of a differential amplifier circuit using an amplifier (AD620, Analog Devices, United States). The two-layered sensor electrode was manufactured by placing a 0.4-mm-diameter gold-plated wire on a metal cloth (1.5 × 2 cm) with conductive silicone containing 4% carbon powder and covered by silicone with 2.6% carbon powder (Togo et al., 2019). As the electrodes were placed on the trunk, problems such as conduction between the electrodes may occur due to sweat. To prevent this, the entire myoelectric sensor was covered with silicone, and the electrodes were placed 3 cm apart.
4. Controller: A microcomputer board (REK−0003, Kyoei Industry, Japan) equipped with a microcomputer (SH72544R, Renesas Electronics Corporation, Japan) was used as the controller, in which the feature quantity was extracted from the myoelectric sensor output, while the motor intention of the user was estimated by the artificial neural network. A specific information processing method will be described later. The mutual communication between the operation teaching device and the controller were based on a Bluetooth module (Bluetooth Mate Silver WRL−12576, SparkFun, USA). An Android tablet (MediaPad T1 7.0, Huawei, China) was used as a motion teaching device, which sends the label information of the movement of the myoelectric shoulder disarticulation prosthesis corresponding to the myoelectric pattern to the controller. Moreover, all subsequent processing of learning and servo motor control were performed on the controller.
5. Hand: The hand we developed was a prosthetic hand with two degrees of freedom that can realize a gripping posture and capable of achieving about 85% of daily living motion (Cipriani et al., 2010) (Figure 1D) (Jing et al., 2014; Hoshikawa et al., 2015). The hand surface was covered by an elastomeric glove (Yabuki et al., 2016), which brings the hand appearance closer to the healthy-side hand, while its friction improves the hand gripping performance. The hand and glove weights were about 130 and 60 g, respectively.

### Control Method

**Figure 2:**
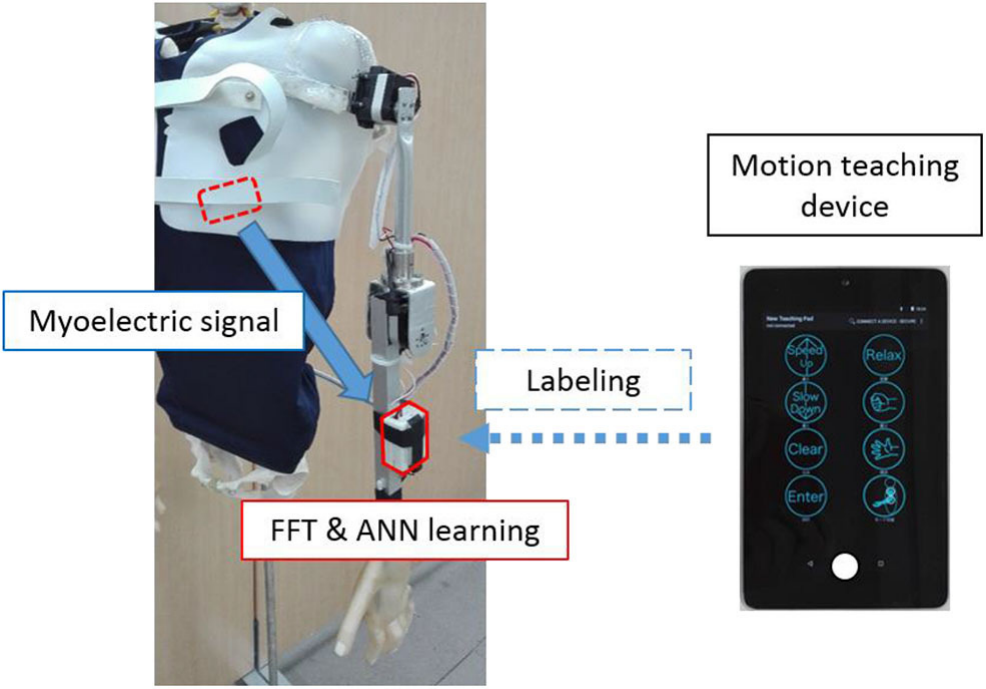
Learning method. The measured myoelectric signals are subjected to frequency analysis on the controller, while the extracted features are used for labeling by the motion learning device.

In this study, the control mechanism of the system was based on a three-layered artificial neural network (ANN), which corresponds to the myoelectric pattern and the motion of the shoulder articulator during user operation (Kato et al., 2006) (Figure 2). First, the motion teaching device was used to label the movements of the user and the shoulder prosthesis in the ANN. On the controller, the EMG signal acquired from the user motion was subjected to fast Fourier transformation to extract the feature vectors of the eight frequency domains. Subsequently, the ANN learns the user motion by using the feature quantities as the teacher signal. The number of neurons in each layer was 24 (3 channel × 8 feature quantity) for the input layer, 32 for the intermediate layer, and 3 for the output layer. The above control method can discriminate operations with kinds more than the number of sensors and can flexibly deal with the mechanical change. Moreover, it has an advantage of reducing malfunction compared to threshold control using amplitude value. From our preliminary experimental results on the training time of the above control system for 5 adult males, the average training time was quite short, as 73.4 seconds (standard deviation was ±21.5). Therefore, once mounted, the proposed system can be used without long-term training.

In this study, the operation was simplified to stabilize the control, such as the degree of operational freedom which was reduced. First, the movements of the shoulder disarticulation prosthesis were classified into four different groups: lifting and lowering the arm (coordinated movement of the shoulder and elbow) and opening and closing the hand. Next, switching operation was provided during operation, allowing four operations to be controlled by two intuitive user motion and one switching signal. Additionally, the hand malfunctioning of opening and closing can be reduced. Particularly, to satisfy these requirements, EMG was measured by attaching two myoelectric sensors to the user trunk and one to the head. However, as the trunk stretches widely, suitable place identification for myoelectric measurement becomes difficult. Therefore, in this study, based on multipoint measurement, we identified the appropriate EMG measurement points in the trunk in advance.

### Identification of Proper EMG Measurement Points of Trunk

Generally, when measuring EMG, the first muscle placement is anatomically checked, followed by consideration of the physique, muscle mass, fat mass, etc., of the subject; finally the electrode is placed in an easily measurable location. However, as the same streaks are widely distributed in the body trunk, finding places for easy EMG measurement is necessary. Therefore, here, to identify proper EMG measurement points of the trunk, multipoint EMG measurement was conducted as a preliminary experiment.

#### Test Subjects

The subjects were five healthy adult males (4 subjects in their 20s and 1 subject in his 50s). As the subject who participated in the evaluation experiment in Chapter 3 was in his 50s, a healthy subject in his 50s was recruited to investigate the influence of age. This experiment was approved by the Ethics Committee of the University of Electro-Communications [management number 10006 (4)] and was conducted with written informed consent from the subject.

#### Experimental Equipment

**Figure 3:**
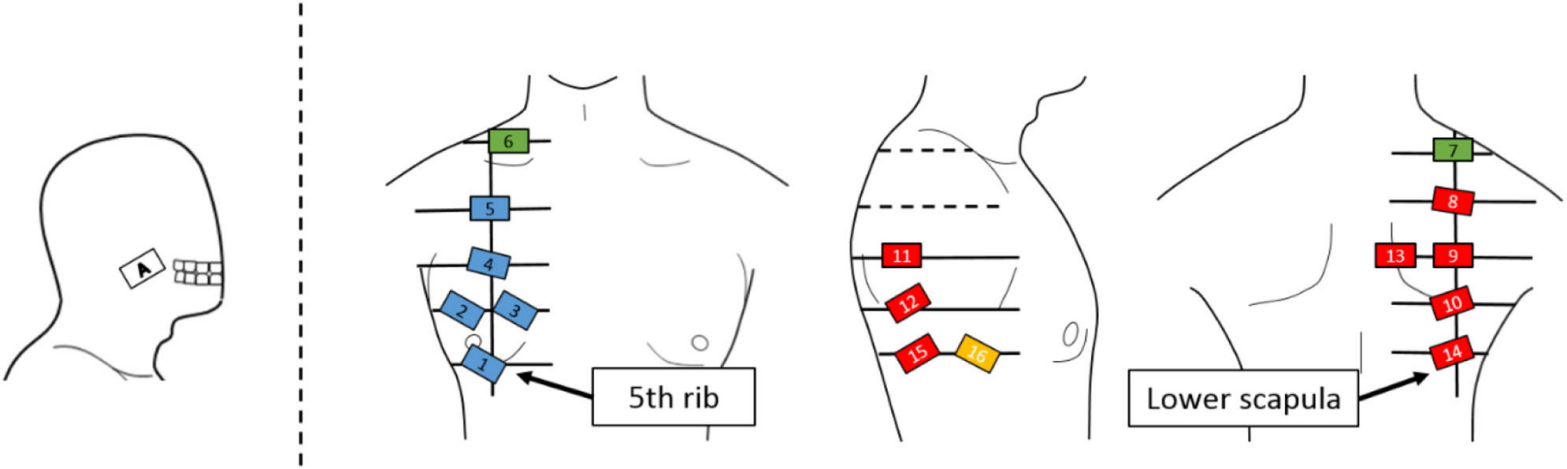
Electrode positions. An EMG sensor is placed on a grid line extending from the lower fifth rib to the lower scapula. The EMG sensor is placed on the cheek where the EMG signal of the masseter muscle can be measured for movement switching.

The sensor electrodes were numbered from 1 to 16 and placed near the right shoulder. Figure 3 depicts the layout of the electrodes, which were arranged in a grid pattern along the muscle fiber direction from the fifth rib to the lower shoulder blade part. Regarding the pasting position, for each body part, 1 to 5 were defined as chest, 6 and 7 as shoulder, 8 to 15 as back, and 16 as side flank. The body ground was measured from the elbow or clavicle. For the myoelectric sensor, a differential amplification circuit (AD 620, Elfo Engineering Co., Ltd, Switzerland) was used, whereas a wet electrode (Biorode electrocardiogram electrode, GE Healthcare, Japan) was employed as an electrode pad for stable EMG measurements. To obtain the data, we used the measurement software C-LOGGER with I/O unit supporting multiple channels (AIO-163202 FX-USB, CONTEC, Japan) and integrated board to connect the unit and electrode. Moreover, the sampling rate was set as 2000 Hz.

#### Experimental Procedure

**Figure 4:**
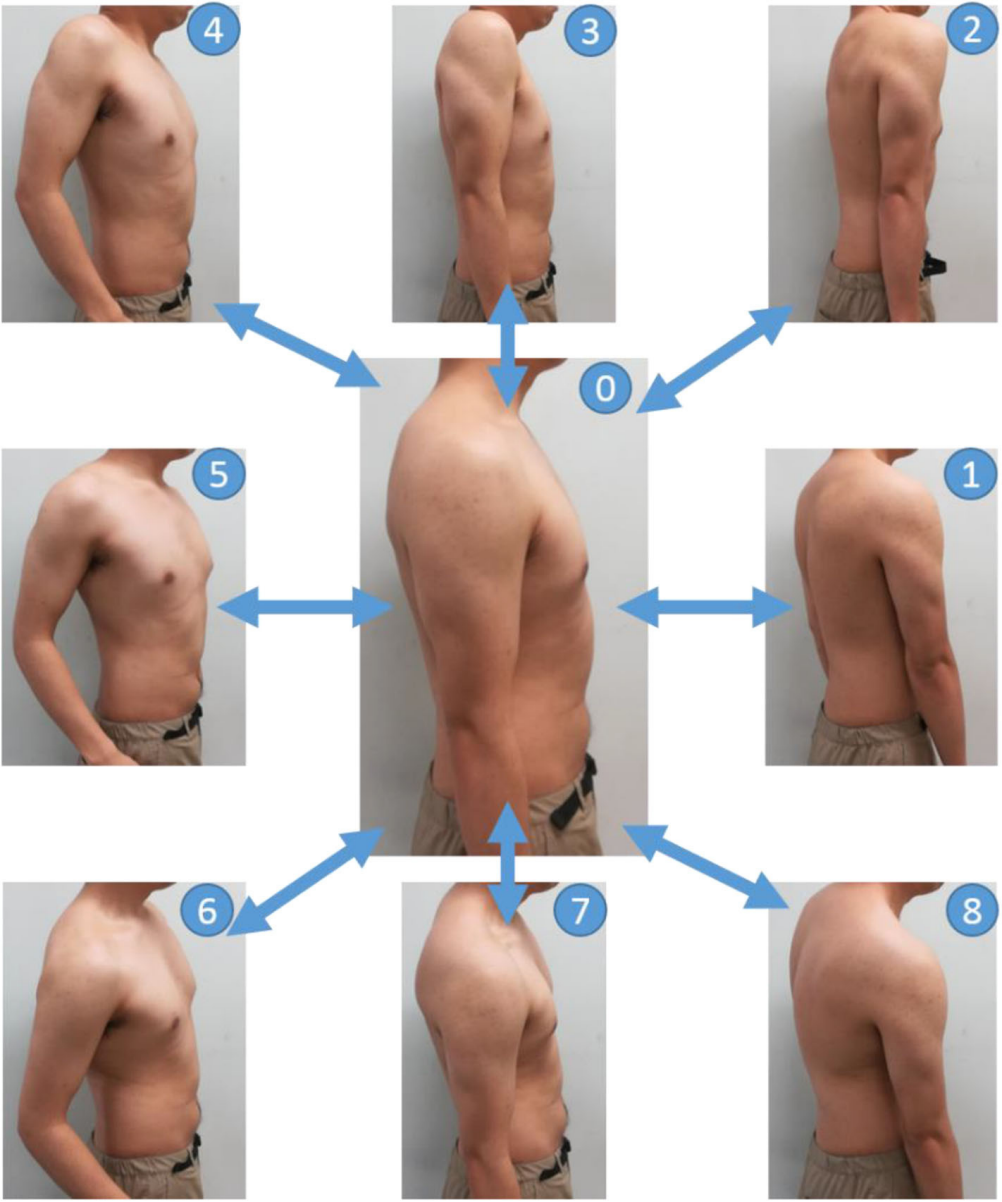
Movements in the EMG measurement experiments. The basic posture is labeled as 0. First, push the shoulder forward from the basic posture and return back to the basic posture (0–1–0). Next, the shoulder is pushed out from the basic posture in a forward and upward oblique direction of 45 degrees and return back to the basic posture (0–2–0). The direction in which the shoulder is pushed out is shifted by 45 degrees, and the operation is continued until the shoulder rotates (0–8–0).

Figure 4 shows the operation for the EMG measurement of the subject shoulder. Muscles near to the shoulder were rarely moved individually. Therefore, some subjects were unaware of how to move the streak near the shoulder. Thus, in this study, we measured the EMG from all the muscles around the shoulder by having the subject perform the motion, shown in Figure 4. Specifically, the shoulder was protruded counterclockwise in each direction at intervals of 45° from the anterior direction, with the resting shoulder (0) in the sagittal plane as the center (0–1, 0–2, …, 0–8), returning action (8 actions) and resting (1 action) to the subject by oral explanation and action. The operation interval was set to 2 s and indicated to the subjects by using a metronome. The measurements of a total of five sets were carried out as one set per round.

#### Analytical Method

In this study, PCA was used as the method to determine the appropriate myoelectric measurement point from the obtained myoelectric data via multipoint measurement (Jolliffe, 2002), and MATLAB was used as the analysis software.

PCA is a multivariate analysis technique that allows the elimination of the correlation of the original data consisting of many variables and the reduction of its feature dimension through the main component variable. Consider the electromyographic measurement data *X* ∈ ℝ consisted of *K* ∈ ℕ channels with a sample size *N* ∈ ℕ as a sample set (*K* = 16 in this study), and assume the average of myoelectric data of a certain channel to be μ ∈ ℝ. The (*i, j*) element *r*_*ij*_ of the covariance matrix *R* ∈ ℝ of the sample set is expressed by the following equation (*i, j* = 1, *K*):

(1) $$\begin{array}{l} r_{ij}=\frac{1}{N-1}\overset{N}{\underset{n=1}{\sum }}\left(X_{in}-\mu_{i}\right)\left(X_{jn}-\mu_{j}\right) \end{array}$$

For the matrix, the eigenvector that satisfies its eigenvalue is the main component, which can be expressed as

(2) $$\begin{array}{l} R\psi =\lambda \psi \end{array}$$

Here, the myoelectric data of 16 dimensions obtained by the multipoint measurement was subjected to PCA, and totally 16 principal components were obtained. The contribution ratio *C*_*m*_ ∈ ℝ of the *m* ∈ ℕ principal component and the cumulative contribution ratio *P*_*m*_ ∈ ℝ up to the *m*-th principal component are expressed by the following expressions:

(3) $$\begin{array}{l} C_{m}=\frac{\lambda_{m}}{tr\left(R\right)} \end{array}$$

(4) $$\begin{array}{l} P_{m}=\overset{m}{\underset{i=1}{\sum }}C_{i}=\frac{\sum_{i=1}^{m}\lambda_{i}}{tr\left(R\right)} \end{array}$$

In this study, the cumulative contribution ratio of each principal component was employed to decide whether the explanation of the original EMG data was more than 80% in two principal components. The principal component loading amount *f* ∈ ℝ of *k* ∈ ℕ ch in the *m*-th principal component is expressed by the following equation:

(5) $$\begin{array}{l} f_{mk}=\frac{\sqrt{\lambda_{m}}\psi_{m}}{r_{kk}} \end{array}$$

Assuming that the electrode position, where the amount of principal component load is the largest, is the proper EMG measurement point *A* ∈ ℝ, the appropriate electromyographic measurement point *A* in the *m*-th principal component is expressed as

(6) $$\begin{array}{l} A_{m}=\underset{k}{\text{argmax}}(f_{mk}) \end{array}$$

Therefore, the electrode position, which most contributes to the two calculated principal components, was specified through the principal component load amount. Meanwhile, the appropriate EMG measurement points were estimated by subjecting myoelectric data obtained by multipoint measurement to PCA.

#### Result of the Preliminary Experiment

**Figure 5:**
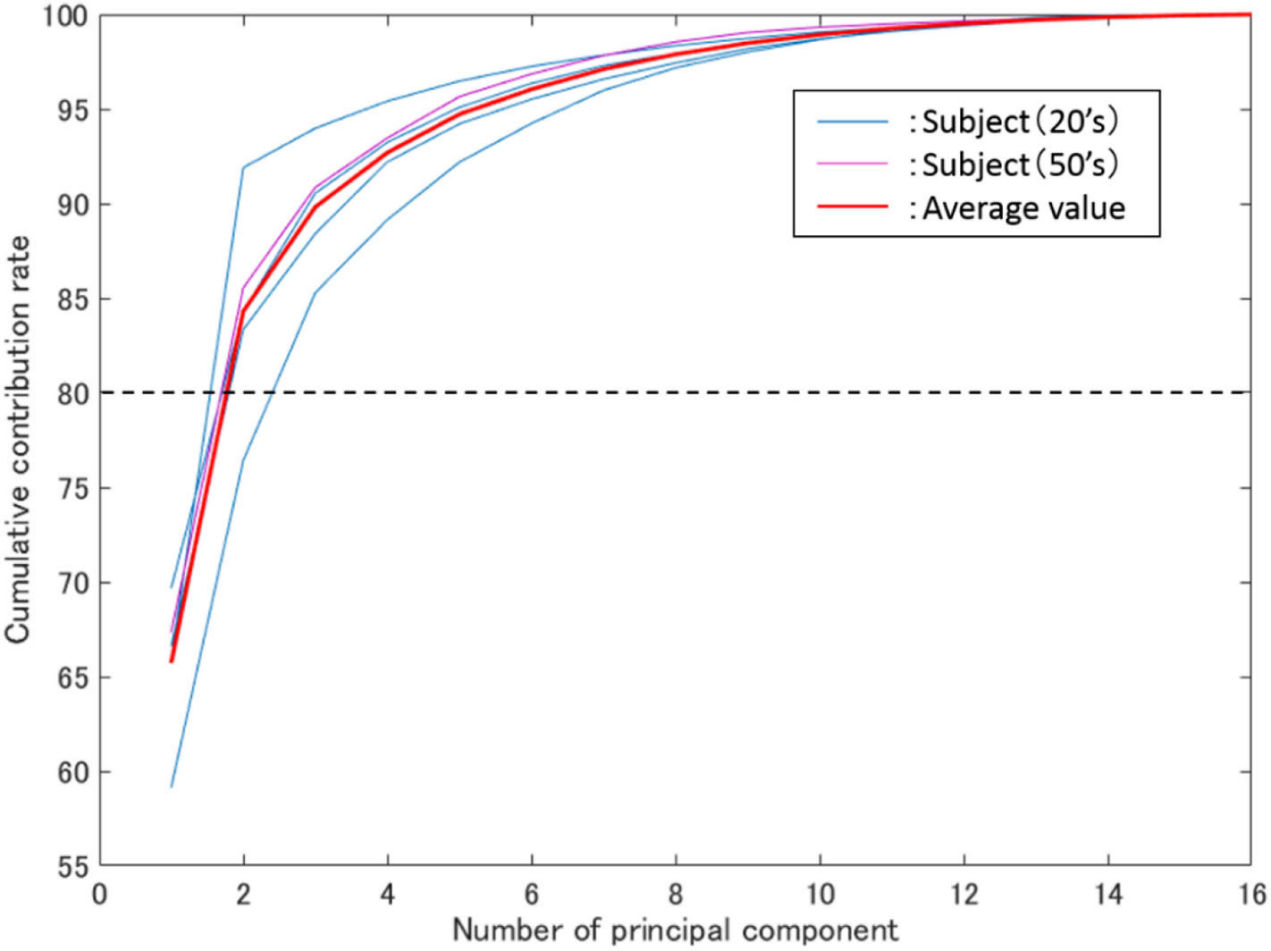
Average cumulative contribution rate. The vertical axis represents the cumulative contribution rate, the horizontal axis represents the number of principal components, while the blue and purple thin lines represent the results of all subjects in their 20 s and 50 s.

**Figure 6:**
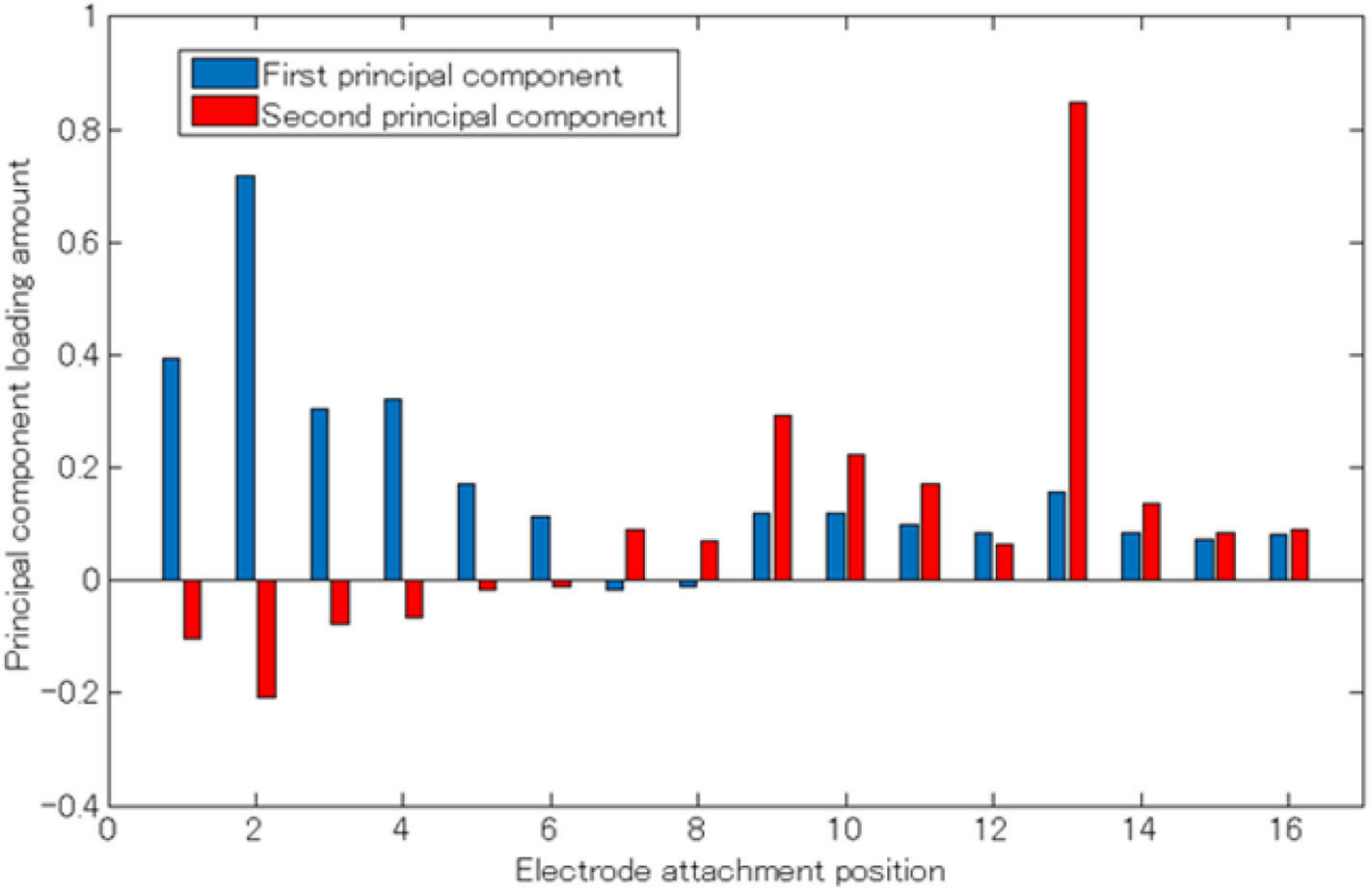
Principal component load (Subject 1). The horizontal axis represents the electrode attaching position, the vertical axis represents the principle component load, and the blue and red bar graphs represent the first and second principle components, respectively.

Figure 5 shows the average cumulative contribution rate of all subjects, while Figure 6 shows a principal component load and electrode attachment position for a typical subject (Subject 1). Moreover, Table 1 summarizes the electrode attachment positions that had the strongest influence on the first and second principal components of all subjects.

**Table 1:** Appropriate EMG measurement points for each subject.

	**1st principal component**	**2nd principal component**
Subject 1	Chest (2)	Back (13)
Subject 2	Chest (2)	Back (12)
Subject 3	Shoulder (6)	Chest (1)
Subject 4	Chest (3)	Shoulder (7)
Subject 5 (50's)	Chest (4)	Back (8)
Chest:1~5	Shoulder:6·7 Back:8~15 Side:16	

*Blue highlights the chest, and red highlights the back*.

In Figure 5, the thin blue, purple, and red lines are the average cumulative contribution rates of the experimental results for 5 trials of subjects in their 20s and 50s, as well as all subjects, respectively. Meanwhile, the two main components were confirmed to have exceeded 80% (average 84%). Additionally, as shown in Figure 5, the cumulative contribution rates of subjects in their 50s and the average of all subjects exhibited close values.

From Figures 3, 6, it can be observed that there existed many electrodes with high contribution ratio in the chest for the first and in the back for the second principal components. Table 1 also demonstrates that this tendency was strong in all subjects. Subjects 3 and 4 also had the third principal component, while the back was the appropriate electromyogram measurement site.

#### Discussion of the Preliminary Experiment

As shown in Figure 5, the average cumulative contributions of subjects in their 20s and 50s were close to each other, suggesting that there exists little difference between ages. Moreover, it can be claimed that as the average value of the cumulative contribution rates of all examinees exceeded 80% by the second principal component, it enables the action explanation of the trunk division. As shown in Figures 3, 6, because the first and second principal components have high contribution rates of the electrodes on the chest and back, the myoelectric of the pectoralis and the back muscles were considered to be dominant in these components. Meanwhile, the results of Table 1 reveal that the control of the shoulder prosthesis was enabled by the EMG of the trunk division of 2 ch, while the chest and back were seemed to be suitable for the electrode arrangement.

Therefore, the shoulder disarticulation prosthesis is considered to be sufficiently controlled by using two myoelectric parts of the chest and back of the trunk. Additionally, the results of the average contribution of all subjects and the cumulative contribution rates of subjects in their 50s demonstrated similar values. Therefore, the abovementioned results could be applied to those with an amputated shoulder in their 50s.

## Experimental Evaluation

Through the appropriate EMG measurement point obtained by the PCA, the operational function of the developed myoelectric shoulder disarticulation prosthesis was evaluated both by healthy and bilateral shoulder disarticulation amputee subjects, in which the subjects gripped and moved a ball in three-dimensional space using our developed system.

### Subject

The subjects were 4 healthy adult males (20s) and bilateral shoulder disarticulation amputee male (50s). This experiment was approved by the Ethics Committee of the University of Electro-Communications [management number 10,006 (4)], and an experiment was conducted after informed consent was obtained from the subject in writing.

### Correspondence Between the EMG Pattern and the Movement of the Shoulder Prosthesis

**Figure 7:**
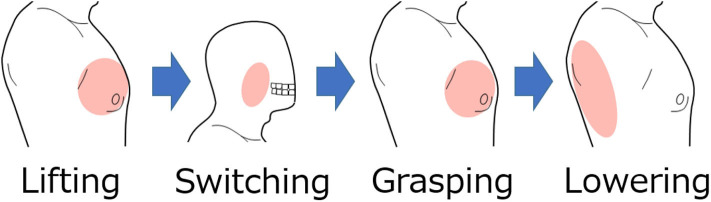
Example of operating procedure for shoulder disarticulation prosthesis. The arm of the shoulder disarticulation prosthesis rises when the shoulder adduction is the system input, while the hand is in gripping motion when the shoulder adduction is the input after the biting motion. In this state, the arm lowers by inputting the shoulder abduction.

The movement of the shoulder disarticulation prosthesis is the lifting/lowering of the arm with coordinated motion of the shoulder and elbow, as well as the grasping/opening motion of the hand. Together with the two places (electrode no. 2 and 13 in Figure 3) of the appropriate EMG measurement part of the trunk identified in the previous chapter, the electrodes were placed in three places, including the cheeks (electrode A in Figure 3), which were used for motion switching. Table 2 shows a correspondence table between the user motion and the movement of the myoelectric shoulder disarticulation prosthesis. As shown in Table 2, the movement of the myoelectric shoulder disarticulation prosthesis mainly corresponds to two motion types of the chest (horizontal adduction of the shoulder) and the back (shoulder horizontal abduction). The contractions of the pectoral and dorsal muscles correspond to the flexion and extension, respectively. Thus, the lifting and closing motion as well as the lowering and opening motion of the shoulder disarticulation prosthesis correspond to the flexion and extension joint motions of the user, respectively. Here, the correspondence in the joint space was considered, while shoulder horizontal adduction was made to correspond to lifting and grasping motions of the shoulder disarticulation prosthesis. Switching between the two motion types is done with a biting (symbol A in Figure 3) motion. As a result, it was possible to perform four operations with motions on the chest and back, which were the proper EMG measurement points. A specific operation example is shown in Figure 7, which demonstrates procedures of lifting the arm, grasping the hand, and lowering the arm. By switching the motion by biting, the movement of the shoulder disarticulation prosthesis is restricted to the grasping or lowering, leading to the hand being kept closed during gripping and, thus, making it possible to prevent accidents, such as dropping an object.

**Table 2:** Movement correspondence pattern of subject and shoulder disarticulation prosthesis.

**Subject motion**	**Motion of shoulder disarticulation prosthesis**
Chest	Lifting or Grasping
Back	Opening or Lowering
Cheek	←Switching motion →

### Experimental Procedure

**Figure 8:**
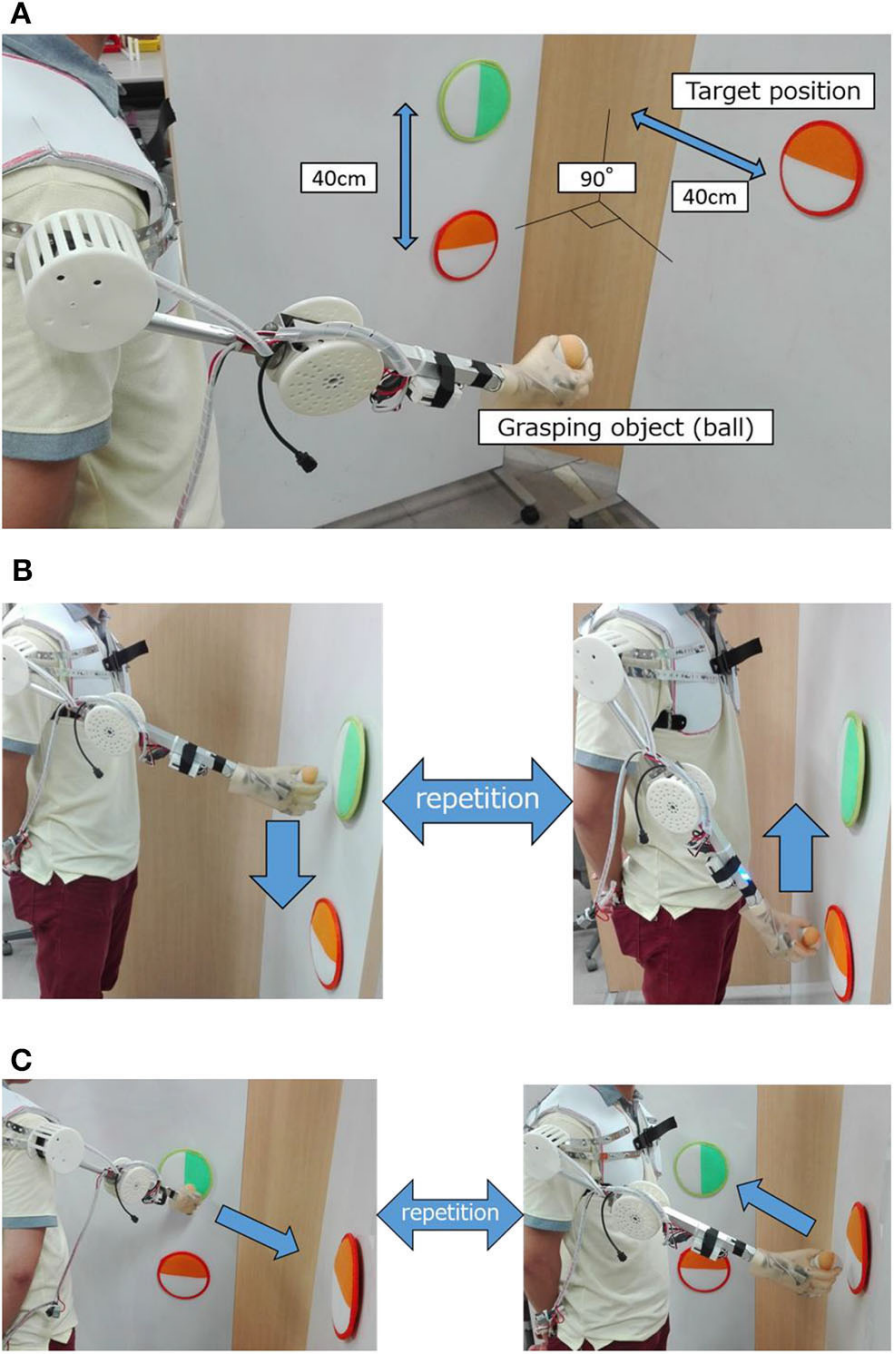
Experimental evaluation environment and experiment operation. **(A)** Experimental environment. **(B)** Experimental operation (vertical motion). **(C)** Experimental operation (horizontal motion).

In the evaluation experiment, subjects gripped and moved a ball within a certain period of time. Figure 8 shows the experimental environment and operation. As shown in Figure 8A, two target positions were vertically arranged with a 40-cm space on the vertical wall surface. The upper target position was adjusted to the chest height of the subject, from which another target position was placed at the same height with the orientation turned 90 degrees to the left. The experimental movements were vertical (Figure 8B) and horizontal (Figure 8C), including grasping and releasing a ball between the arranged target positions. The experimental time was 1 minute per trial; there were 5 trials for each operation.

### Results and Discussion of the Evaluation Experiment

**Figure 9:**
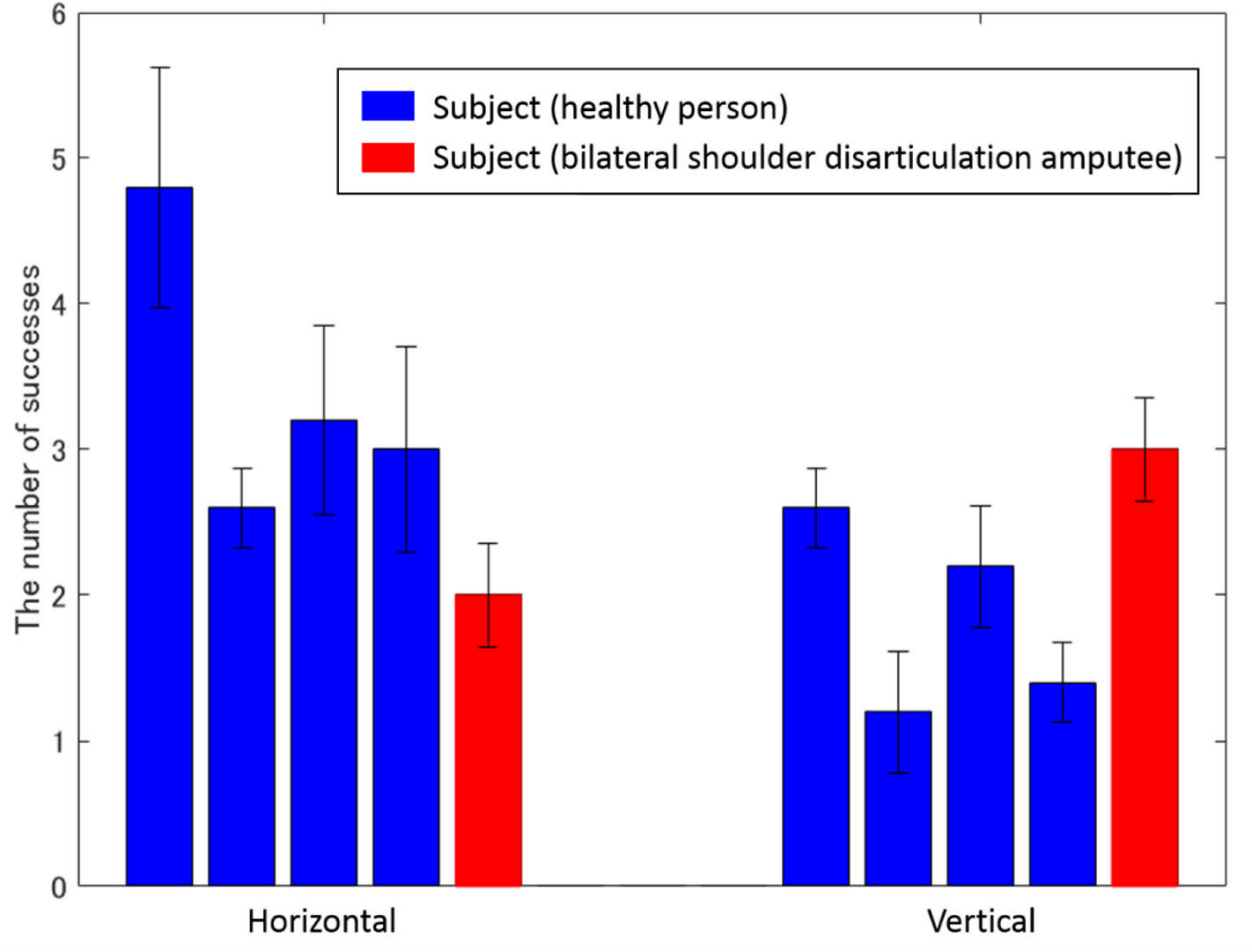
Average number of successes for each subject. The left and right bars represent vertical and horizontal movements, while the vertical axis represents the number of successes. Blue and red bars show the experimental results of healthy and bilateral shoulder disarticulation amputee subjects, respectively.

Figure 9 shows the average number of successful movements of all subjects, including the bilateral shoulder disarticulation amputee (number of times the subjects grabbed the ball, removed and transferred it to the other target, and released the hand). As shown in Figure 9, all subjects performed more than 1 successful motion as a trial average in vertical transfer (average of all subjects was 2.08 times) and more than 2 successful motions as a trial average in horizontal transfer (the average of all subjects was 3.12 times).

**Figure 10:**
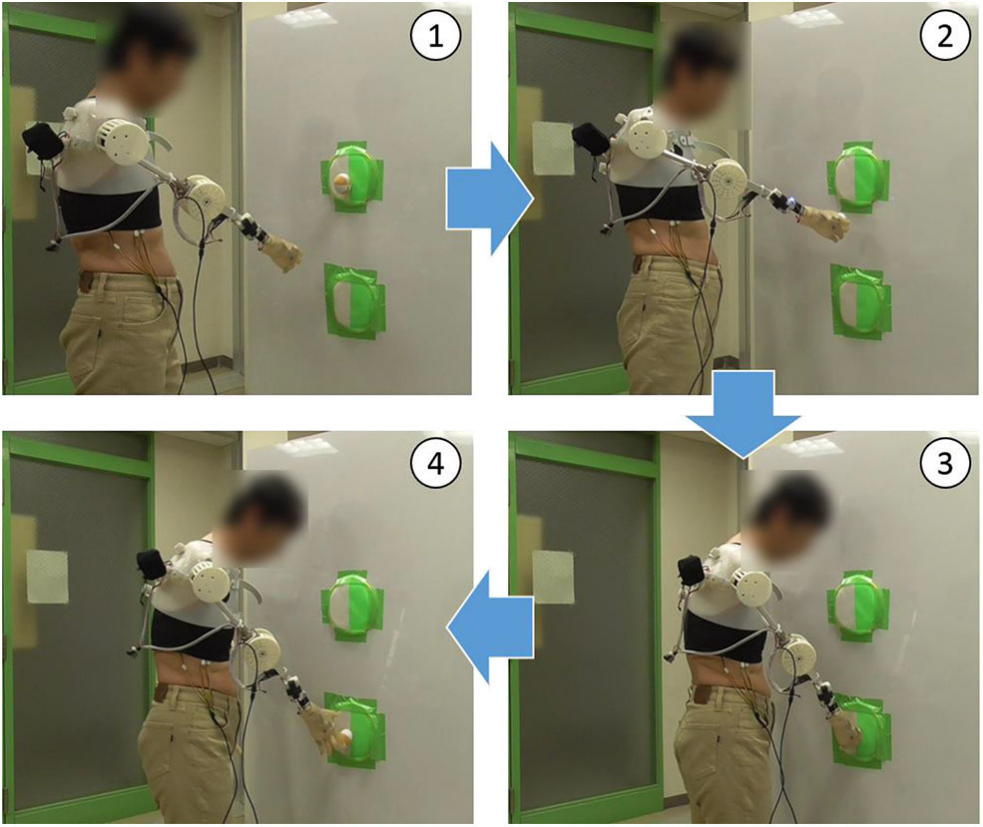
Evaluation experiment by bilateral shoulder disarticulation amputee. (1) Lifting arm. (2) Taking the ball out of the target and lowering arm. (3) Putting the ball in the target. (4) Releasing the ball from the hand.

As shown in Figure 9, all subjects successfully operated the shoulder disarticulation prosthesis with EMG control using appropriate EMG measurement points. As shown in Figure 10, the bilateral shoulder disarticulation amputee also successfully carried out the same experiment as healthy persons. In an earlier experiment (on the control of the shoulder disarticulation prosthesis using the TMR operation Laura et al., 2008), a resting period of 9 months and a practice time of 100 h were required after the operation. Meanwhile, our results showed that the proposed shoulder disarticulation prosthesis system was quickly able to learn the correspondence between the motor intention of the user and the movement of the shoulder disarticulation prosthesis through an appropriate EMG measurement point of the trunk.

Laura et al. conducted the experiments in which subjects who underwent TMR surgery grasped and moved an object in a vertical direction using a three-degrees-of-freedom prosthesis and a six-degrees-of-freedom prosthesis (Laura et al., 2008). They reported that the time required to move three objects with each prosthesis was 79.2 ± 14.3 seconds for the three-degrees-of-freedom prosthesis and 58.0 ± 9.2 seconds for the six-degrees-of-freedom prosthesis. In comparison, our results showed the subjects with bilateral shoulder dissections succeeded in holding and moving an average of three times in 60 s. These results show that the performance of the shoulder disarticulation prosthesis system developed in this study is comparable to that of the six-degrees-of-freedom prosthesis controlled by TMR surgery without long-term training.

## Conclusion

In this study, we developed intuitively operational powered shoulder disarticulation prosthesis with four degrees of freedom using surface electromyogram control. Moreover, we identified the most suitable place to measure the EMG used to control the shoulder disarticulation prosthesis from the trunk by multipoint measurement. We conducted evaluation experiments of the proposed myoelectric shoulder disarticulation prosthesis using myoelectric at a suitable place. As a result, we identified two proper EMG measurement points from the body trunk being as chest and back and demonstrated by the evaluation experiment that all subjects, including actual bilateral shoulder disarticulation amputee, could manipulate the proposed shoulder disarticulation prosthesis. Thus, we could develop a myoelectric shoulder disarticulation prosthesis using the trunk myoelectric without long-term training. Meanwhile, implementing the function of grasping and moving the target object was also possible. The future work includes increasing the number of sensors, freedom degrees of arm, and the motion numbers that can be obtained while maintaining the simplicity achieved in this study.

## Data Availability Statement

All datasets generated for this study are included in the article/supplementary material.

## Ethics Statement

The studies involving human participants were reviewed and approved by the Ethics Committee of the University of Electro-Communications. The patients/participants provided their written informed consent to participate in this study. Written informed consent was obtained from the individual(s) for the publication of any potentially identifiable images or data included in this article.

## Author Contributions

SK, YT, ST, YJ, and HY: conceived and designed the experiments. SK, YT, and ST: performed the experiments. SK and ST: analyzed the data. SK, ST, YJ, and HY: wrote the paper. All authors gave final approval for publication.

## Conflict of Interest

The authors declare that the research was conducted in the absence of any commercial or financial relationships that could be construed as a potential conflict of interest.
